# Impact of facial telangiectasias and anemia on quality of life in patients with hereditary hemorrhagic telangiectasia: a cross-sectional study using FACE-Q

**DOI:** 10.1186/s41687-026-01179-x

**Published:** 2026-08-26

**Authors:** Nadia Sadok, Franziska Bielefeld, Carl Nikola Martin, Eva-Marie Skoda, Katharina Klinger, Felicia Toppe, Marie Carolin Schleupner, Lukas Boosfeld, Björn Kropf, Noemi Voß, Antonia Lakomek, Anna-Lena Valina, Christina Kaiser, Julia Garvert, Stephan Lang, Freya Droege

**Affiliations:** 1 Department of Otorhinolaryngology, Head and Neck Surgery, Essen University Hospital, and VASCERN HHT Reference Center, University of Duisburg-Essen, Hufelandstrasse 55, 45122 Essen, Germany; 2 LVR University Hospital Essen, Clinic for Psychosomatic Medicine and Psychotherapy, Clinics and Institute of the University of Duisburg-Essen, University of Duisburg-Essen, Essen, North Rhine-Westphalia Germany

**Keywords:** Hereditary hemorrhagic telangiectasia, Anemia, Quality of life, Patient reported outcome measures, Face, Patient satisfaction

## Abstract

**Background:**

Hereditary hemorrhagic telangiectasia (HHT) is an autosomal dominant disorder characterized by telangiectasias and visceral arteriovenous malformations. While the clinical manifestations are well described, its psychosocial impact—particularly with regard to visible facial telangiectasias and anemia-related appearance changes—remains insufficiently studied. This study aimed to assess appearance-related quality of life in individuals with hereditary hemorrhagic telangiectasia.

**Methods:**

In this cross-sectional, single-center study, adults with hereditary hemorrhagic telangiectasia were recruited at a specialized HHT center between November 2023 and May 2024. Participants completed validated patient-reported outcome measures, including the Facial Assessment and Cosmetic Enhancement (FACE-Q) questionnaire and the Patient-Reported Outcomes Measurement Information System (PROMIS-29 +2) profile. Besides that, a standardized questionnaire assessing demographic and clinical characteristics, subjective perceptions of pallor, and cosmetic disturbance was administrated. Disease severity was assessed using the Epistaxis Severity Score and the Toronto Severity Index. Analyses were performed for the overall cohort and stratified by sex to explore potential sex differences in health-related quality of life and symptom perception. Multivariate linear regression analyses were performed to identify independent predictors of FACE-Q subscale outcomes.

**Results:**

A total of 116 participants with HHT were included (mean age 60 ± 14 years; 62% were female). Subjective appearance perception showed strong effects: perceiving pallor or tiredness due to low hemoglobin levels was associated with greater appearance-related psychological distress (adjusted β = −9.09, 95% CI −17.23 to −0.94), whereas perceiving facial telangiectasias as cosmetically disturbing was associated with multiple appearance-related outcomes, including lower social function (adjusted β = −9.51, 95% CI −18.04 to −0.99), greater appearance-related psychological distress (adjusted β = −9.33, 95% CI −16.51 to −2.16), and lower satisfaction with the nose (adjusted β = −17.39, 95% CI −28.94 to −5.84). In contrast, objective clinical parameters and overall disease severity assessed by the Epistaxis Severity Score and the Toronto Severity Index were not independently associated with appearance-related quality of life.

**Conclusions:**

In HHT, appearance-related quality of life is driven predominantly by subjective perception and psychosocial factors.

**Supplementary Information:**

The online version contains supplementary material available at https://doi.org/10.1186/s41687-026-01179-x.

## Background

Hereditary hemorrhagic telangiectasia (HHT), also known as Osler-Weber-Rendu syndrome, is a rare autosomal dominant vascular disorder characterized by mucocutaneous telangiectasias and arteriovenous malformations (AVMs) affecting multiple organs [1–6]. Recurrent epistaxis and gastrointestinal bleeding frequently lead to chronic iron deficiency and anemia, while visible facial telangiectasias may cause cosmetic concerns.

While the physical and genetic characteristics of HHT are well described in the literature, less attention has been given to its psychosocial impact, particularly the effects of visible lesions such as facial telangiectasias [7–10]. Studies using the FACE-Q have shown that satisfaction with facial appearance and appearance-related distress differ between sexes in the general population, with women reporting lower satisfaction with facial appearance and greater appearance-related distress than men [11]. Although these findings originate primarily from normative and cosmetic populations and are limited by the underrepresentation of men, they suggest that sex may influence patient-reported perceptions of facial appearance and should therefore be considered when evaluating appearance-related outcomes in HHT [11–13]. These lesions may cause substantial cosmetic concerns, social embarrassment, and psychological distress. In parallel, chronic anemia accompanied by visible pale skin may cause persistent fatigue, reduce physical capacity [14], and diminish overall Quality of Life (QoL) [15]. The unpredictable course of HHT and the requirement for frequent medical interventions further increase psychological burden. Addressing the comprehensive impact of HHT requires an approach that incorporates both clinical management and support for patients’ emotional and social well-being. However, the relationship between physical symptoms and patient-reported outcomes is insufficiently explored in the current literature and requires further investigation. Moreover, given evidence from FACE-Q studies indicating sex-related differences in appearance-related patient-reported outcomes, as well as potential sex-based differences in hemoglobin thresholds and iron metabolism and evidence suggesting that anemia and symptom burden may influence QoL differently by sex [15, 16], these associations should be systematically evaluated in HHT. For that reason, this study aims to assess QoL and patient satisfaction in individuals with HHT, focusing on the roles of facial telangiectasias and anemia, including self-perceived paleness, and exploring potential sex-based differences in these associations. We hypothesize that these factors are independently associated with decreased QoL and satisfaction in HHT, as measured by patient-reported outcome measurements.

## Methods

### Study design & participants

This cross-sectional observational single-center study included adult patients diagnosed with HHT according to the Curaçao criteria (≥3) [2], and/or genetic testing. Participants were recruited from the West German HHT center between November 2023 and May 2024, specifically those who had visited within the previous five years and met the study’s eligibility requirements. Study information was distributed via mailed letters, and prepaid envelopes were provided to facilitate the return of completed questionnaires and signed consent forms. Additionally, patients attending the clinic during the study period were recruited. Identical questionnaires were administered, with supplementary questions to collect information on the Curaçao criteria or genetic testing to confirm the diagnosis of HHT. The study received approval from the Medical Ethics Committee of the University Hospital Essen (approval number: 23-11519 BO) and registered at the clinicaltrials.gov: NCT06261333.

### Procedure

A standardized questionnaire was administered to obtain data on participant characteristics (see supplementary Figure 1). Comprehensive demographic and clinical data related to HHT were systematically collected and securely stored in an electronic database.

### Determinants and definitions

Age was recorded in years, and the Body Mass Index (BMI) was calculated as weight in kilograms divided by the square of height in meters (kg/m^2^). Smoking status was classified as “yes” or “no.” Anemia was defined according to WHO criteria as hemoglobin levels below 12 g/dl in women and 13 g/dl in men [16]. Comorbidities including psychiatric comorbidities were self-reported and coded as “yes” or “no.” According to the participant’s self-report professions were classified into three categories: occupations with customer contact, without customer contact, or retired. Relationship status was documented as single or in a relationship. Facial telangiectasias were assessed by patient self-report, and the presence of facial telangiectasias was recorded as “yes” or “no”. Participants with telangiectasias were asked about previous laser treatment. The degree of disability (Grad der Behinderung, GdB) was recorded as an indicator of global disease-related impairment. The GdB is a standardized German metric that quantifies the overall impact of chronic disease on functional capacity and daily life, ranging from 20 to 100, with higher scores indicating more severe disability [17]. GdB was categorized as none (GdB < 20), mild (GdB 20–30), moderate (GdB 40–50), or severe (GdB ≥ 60).

### Questionnaires & scoring

**FACE-Q:** The primary instrument used for evaluating patient-reported outcomes was the Facial Assessment and Cosmetic Enhancement Quality of Life Questionnaire (FACE-Q). This questionnaire is specifically designed to assess satisfaction with facial appearance. The FACE-Q comprises separate subscales: Social function, Psychological function, Satisfaction with Facial Appearance Overall, Appearance-related psychological distress, Satisfaction with nostrils, Satisfaction with nose. Each subscale is scored independently, rather than providing an overall score. Subscale scores are calculated by summing the raw item scores, with missing data imputed using the within-person mean if fewer than 50% of items are missing. Final scores were transformed using conversion tables and range from 0 (worst) to 100 (best) [18–20].

**PROMIS 29 + 2:** The Patient-Reported Outcomes Measurement Information System (PROMIS-29 +2) Profile v2.1 is an enhanced version of the original PROMIS-29 instrument. It assesses health-related quality of life (HRQoL) across eight core domains: Physical Function, Anxiety, Depression, Fatigue, Sleep Disturbance, Pain Interference, Pain Intensity, and Ability to Participate in Social Roles and Activities. In addition, two items from the Cognitive Function – Abilities v2.0 instrument are included to provide a broader assessment of cognitive health. PROMIS T-scores are standardized to a reference population with a mean of 50 and a standard deviation of 10. Higher scores indicate better functioning in positively oriented domains (e.g., Physical Function, Ability to Participate in Social Roles and Activities) and greater symptom burden in negatively oriented domains (e.g., Anxiety, Depression, Fatigue, Sleep Disturbance, Pain Interference). Pain intensity was assessed using the single-item PROMIS pain intensity measure on an 11-point numeric rating scale ranging from 0 (no pain) to 10 (worst imaginable pain). Raw scores were used for analysis in accordance with PROMIS scoring guidelines. Typical scores range from 20 to 80, with deviations from the mean indicating better or worse outcomes compared to the general population [21].

**Epistaxis severity score (ESS):** The ESS is the primary patient-reported metric for evaluating nosebleeds associated with HHT. Nosebleed severity is classified as follows: 1 to 4 represents mild severity, 4 to 7 corresponds to moderate severity, and 7 to 10 denotes severe epistaxis [22].

**Toronto Severity Index/ HHT score:** Disease severity was evaluated using the Toronto Severity Index (TSI), a validated composite measure of the clinical burden of HHT. The TSI comprises three domains: chronic bleeding, visceral vascular malformations (VMs), and severe organ involvement. The TSI was calculated only for patients with complete data for all required variables. Patients with pending diagnostic evaluations, specifically those with pending ultrasound assessments for pulmonary or hepatic VMs, were excluded from TSI calculation. In these cases, the TSI was coded as missing. Disease severity was categorized as mild (TSI 0–2), moderate (TSI 3–4), or severe (TSI 5–7) for descriptive and comparative analyses [23].

### Statistical analyses

Descriptive statistics were applied to summarize participants’ characteristics. Continuous variables were reported as means and standard deviations (SD) for normally distributed data, and as medians and interquartile ranges for non-normally distributed data. Nominal variables were described by counts and proportions. FACE-Q scores were presented as means ± SD. To assess factors associated with disability severity, odds ratios (ORs) with 95% confidence intervals (95%-CIs) were calculated using logistic regression models. Analyses were conducted for the overall cohort and stratified by sex to investigate the potential sex differences in health-related QoL and symptom perception. Univariate and multivariate linear regression analyses were performed to identify factors influencing the FACE-Q subscales. In this way, β coefficients and 95%-CIs were estimated. For the regression analyses, both objective and patient-reported determinants were included. Objective measures comprised age, gender, BMI, hemoglobin levels, anemia status, ESS, and TSI. Patient-reported measures included smoking status, profession, relationship status, comorbidity, psychiatric comorbidity, self-perceived pallor or tiredness due to low Hb, perceived cosmetic disturbance caused by facial telangiectasias, and the PROMIS domains. The presence of facial telangiectasias was based on self-report and was not clinically confirmed. Univariate analyses were first conducted for each FACE-Q subscale using these variables. Variables with *p*-values ≤ 0.1 were included in the multivariate model, while those with *p*-values > 0.1 were excluded. In the final model, *p*-values less than 0.05 were considered statistically significant. All statistical analyses were performed using IBM SPSS Statistics for Windows (Version 29.0). Armonk, NY: IBM Corp.

## Results

### Comparison between women and men

**Fig. 1:**
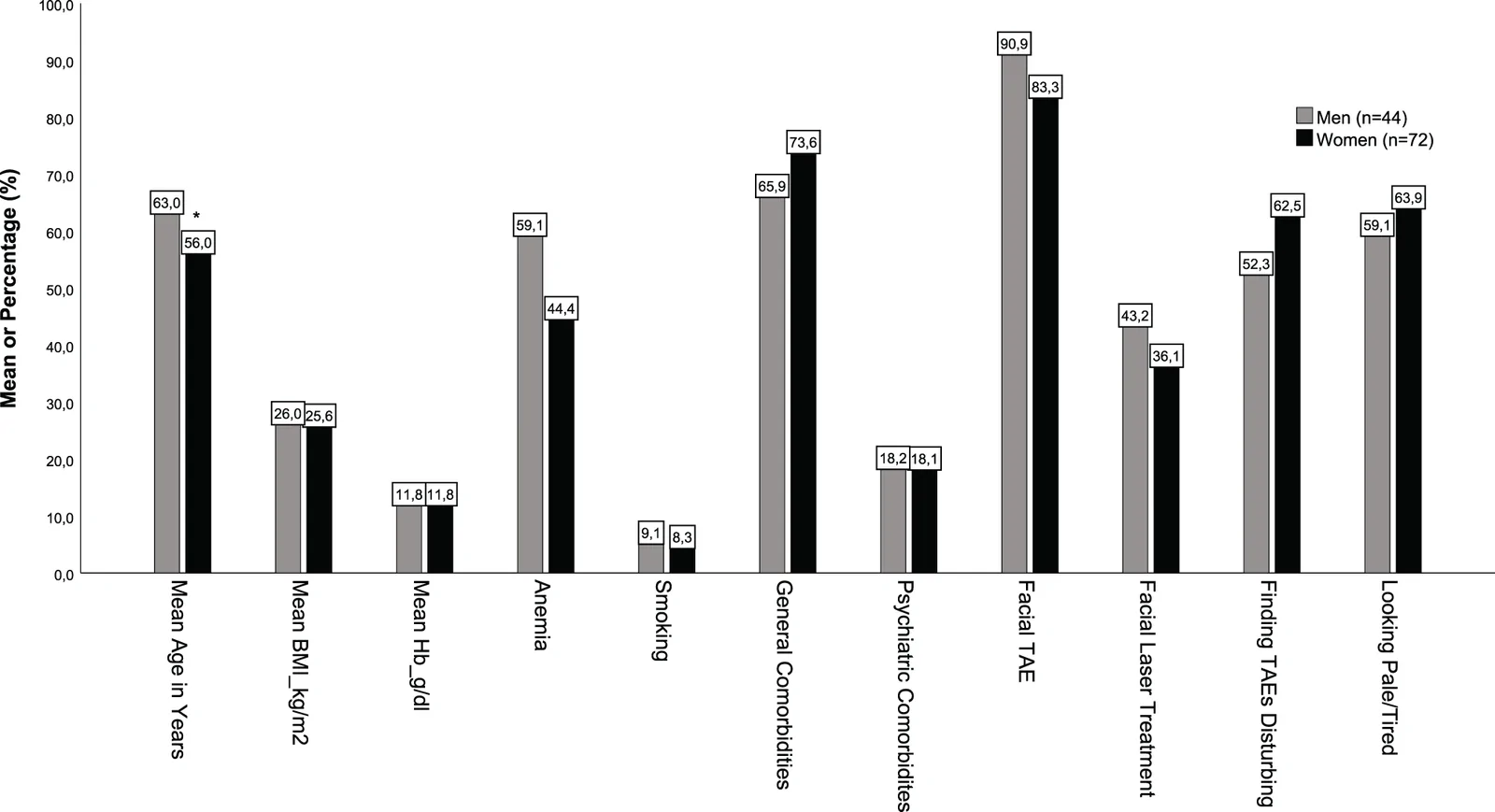
Clinical characteristics of male and female patients with HHT. Footnote: data is shown as mean value and prevalences (%) of clinical characteristics in hereditary hemorrhagic telangiectasia (HHT). *female patients were significantly younger than males (*p* = 0.007); no further significant differences were seen. Number of patients: 116. BMI = body mass index (kg/m^2^); Hb = hemoglobin levels (g/dL); continuous variables (age, BMI, Hb) are presented as mean values; all other variables are categorical and reported as percentages (%). Anemia was defined using Hb cut-offs (<12 g/dL in women, <13 g/dL in men). Smoking was coded as yes/no. General comorbidities refer to conditions other than HHT (yes/no); psychiatric comorbidities indicate current psychiatric disorders (yes/no). Facial telangiectasias denotes patient-reported facial telangiectasia (yes/no); “disturbing” reflects perceived aesthetic burden (yes/no); laser treatment indicates prior treatment of facial telangiectasias (yes/no); pale/tired reflects self-perceived pallor or fatigue due to low Hb (yes/no). Missing data: BMI 2 (1.7%), Smoking 2 (1.7%), psychiatric comorbidities 1 (0.9%), Hb 5 (4.3%), anemia 5 (4.3%), facial telangiectasias 2 (1.7%), laser treatment 15 (12.9%), Pale/tired 8 (6.9%), disturbing 16 (13.8%); none for age or general comorbidities

A total of 116 patients with HHT were included, of whom 11% (13/116) were recruited in person and 89% (103/116) via postal questionnaire. The cohort comprised 72 women (62%) and 44 men (38%). Most demographic and clinical characteristics were comparable between sexes (Table 1; Fig. 1). Women were significantly younger than men (56 ± 13 vs. 63 ± 14 years; *p* = 0.007), whereas no significant differences were observed for BMI, hemoglobin levels, smoking status, comorbidities, disease severity indices (ESS, TSI), or treatment history for facial telangiectasia (*p* > 0.05). Perceived pallor or fatigue related to low hemoglobin levels were reported by 64% of women and 59% of men (*p* = 0.575). Similarly, a higher proportion of women considered their telangiectasias disturbing or unattractive (*N* = 45/72, 63% vs. *N* = 23/44, 53%), although this did not reach statistical significance (*p* = 0.066). Given the absence of relevant sex differences beyond age, subsequent analyses were conducted in the overall cohort.

**Table 1:** Participants characteristics

Characteristics		Total group (N = 116)	Women (N = 72)	Men (N = 44)	p-value
Age [years]	Mean (SD) Missing, N (%)	59 (14) 0 (0)	56 (13) 0(0)	63 (14) 0(0)	*0.007*
BMI [kg/m2]	Mean (SD) Missing, N (%)	26.0 (6.1) 2 (1.7)	26.2 (7.1) 2 (2.8)	25.6 (3.8) 0(0)	0.430
Smoking	Yes, N (%) No, N (%) Missing, N (%)	10 (8.6) 104 (89.7) 2 (1.7)	6 (8.3) 64 (88.9) 2 (2.8)	4 (9.1) 40 (90.9) 0(0)	0.924
Profession/Occupational status*	With customer contact, N(%) Without customer contact, N(%) Pensioner, N (%) Missing, N (%)	41 (35.3) 20 (17.2) 52 (44.8) 3 (2.6)	29 (40.3) 14 (19.4) 27 (37.5) 2 (2.8)	12 (27.3) 6 (13.6) 25 (56.8) 1 (2.3)	0.228
Relationship status	Single, N (%) In a relationship, N (%) Missing, N (%)	15 (12.9) 94 (81.0) 7 (6.0)	8 (11.1) 59 (81.9) 5 (6.9)	7 (15.9) 35 (79.5) 2 (4.5)	0.490
Comorbidities*	Yes, N (%) No, N (%)	82 (70.7) 34 (29.3)	53 (73.6) 19 (26.4)	65.9) 15 (34.1)	0.377
Recognized degree of disability	Yes, N (%) No, N (%) Missing, N (%)	51 (44.0) 64 (55.2) 1 (0.9)	31 (43.1) 40 (55.6) 1 (1.4)	20 (45.5) 24 (54.5) 0(0)	0.851
Categories for degree of disability	<20 (none), N (%) 20–30 (mild), N (%) 40–50 (moderate), N (%) ≥60 (severe), N (%)	4 (3.4) 7 (6.0) 31 (26.7) 10 (8.6)	3 (4.2) 3 (4.2) 19 (26.4) 7 (9.7)	1 (2.3) 4 (9.1) 12 (27.3) 3 (6.8)	0.798
Psychiatric comorbidity	Yes, N (%) No, N (%) Missing, N (%)	21 (18.1) 94 (81.0) 1 (0.9)	13 (18.1) 71 (98.6) 1 (1.4)	8 (18.2) 36 (81.8) 0(0)	0.986
Hemoglobulin level [g/dl]	Mean (SD) Missing, N (%)	11.8 (2.6) 5 (4.3)	11.8 (2.3) 1 (1.4)	11.8 (3.1) 4 (9.1)	0.073
Anemia	Yes, N (%) No, N (%) Missing, N (%)	58 (50.0) 53 (45.7) 5 (4.3)	32 (44.4) 38 (52.8) 2 (2.8)	26 (59.1) 15 (34.1) 3 (6.8)	0.112
Presence of facial telangiectasia	Yes, N (%) No, N (%) Missing, N (%)	100 (86.2) 14 (12.1) 2 (1.7)	60 (83.3) 10 (13.9) 2 (2.8)	40 (90.9) 4 (9.1) 0(0)	0.402
History of laser treatment for facial telangiectasia	Yes, N (%) No, N (%) Missing, N (%)	45 (38.8) 56 (48.3) 15 (12.9)	26 (36.1) 36 (50.0) 10 (13.9)	19 (43.2) 20 (45.5) 5 (11.4)	0.504
Epistaxis Severity Scale	None, N (%) Mild, N (%) Moderate, N (%) Severe, N (%) Missing, N (%)	6 (5.2) 29 (25.0) 44 (37.9) 29 (25.0) 8 (6.9)	4 (5.6) 20 (27.8) 22 (30.6) 20 (27.8) 6 (8.3)	2 (4.5) 9 (20.5) 22 (50.0) 9 (20.5) 2 (4.5)	0.880
Toronto Severity Index	Mild, N (%) Moderate, N (%) Severe, N (%) Missing, N (%)	63 (64) 27 (28) 8 (8) 18 (16)	40 (55.6) 17 (23.6) 5 (6.9) 10 (13.9)	21 (47.7) 10 (22.7) 4 (9.1) 7 (15.9)	0.916
Do you think you look pale or tired due to your low hemoglobin level?	Yes, N (%) No, N (%) Missing, N (%)	72 (62.1) 36 (31.0) 8 (6.9)	46 (63.9) 21 (29.2) 5 (6.9)	26 (59.1) 15 (34.1) 3 (6.8)	0.575
Do you consider them disturbing or unattractive?	Yes, N (%) No, N (%) Missing, N (%)	68 (58.6) 32 (27.6) 16 (13.8)	45 (62.5) 15 (20.8) 12 (16.7)	23 (52.3) 17 (38.6) 4 (9.1)	0.066
PROMIS 29 + 2 domains Physical Function Anxiety Depression Fatigue Sleep Disturbance Ability to Participate in Social Roles and Activities Pain Interference Cognitive Function Pain Intensity on a scale of 0–10)	Mean (SD)	45.2 (8.9) 56.7 (8.0) 56.3 (8.2) 56.0 (9.8) 53.7 (8.6) 45.6 (8.9) 53.0 (9.2) 49.6 (7.3) 2.8 (2.4)	44.8 (8.8) 57.8 (6.9) 57.2 (7.8) 56.6 (9.6) 54.2 (8.9) 45.9 (8.5) 53.7 (9.4) 49.9 (6.8) 3.1 (2.5)	45.8 (9.1) 54.9 (9.4) 55.0 (8.7) 55.0 (10.1) 52.8 (8.2) 45.2 (9.5) 51.9 (9.0) 49.0 (8.1) 2.3 (2.1)	0.693 0.096 0.263 0.467 0.424 0.521 0.426 0.624 0.132

Table 1 presents demographic and clinical characteristics of the study population overall and stratified by sex. Anemia was diagnosed based on hemoglobin levels: women < 12 g/dl, men < 13 g/dl. Values are reported as mean ± standard deviation (SD), median (interquartile range), or number/total number (N) and percentages (%). With the exception of age, characteristics were largely comparable between women and men

Abbreviations: HHT = hereditary hemorrhagic telangiectasia; BMI = body mass index; ESS = Epistaxis Severity Scale; TSI = Toronto Severity Index, PROMIS = The Patient-Reported Outcomes Measurement Information System

Continuous variables were compared using the Mann–Whitney U test or independent-samples t test, as appropriate based on data distribution. Categorical variables were compared using the chi-square test or Fisher’s exact test, as appropriate. A two-sided *p* value < 0.05 was considered statistically significant. *p*-values below 0.05 were marked in italic. *p*-values refer to comparisons between women and men

The TSI was available for most participants (*N* = 98/116 = 85%). In 18 participants (16%), the TSI could not be calculated due to incomplete diagnostic evaluation, most commonly because of pending ultrasound examinations. Specifically, echocardiography was pending in 7 patients (6%), and hepatic ultrasound imaging was pending in 14% of the participants (*N* = 16/116)

Customer-facing occupation was defined according to the participant’s self-report of whether their job involved regular customer or patient contact and was not assigned based on the occupational title

*For detailed information regarding the comorbidities please see Table 2 and for more information regarding the occupational status please see supplemental table S1

**Table 2:** Comorbidities reported in addition to hereditary hemorrhagic telangiectasia

Disease category	n (%)
Cardiovascular diseases (hypertension, coronary artery disease, heart failure, arrhythmia, stroke)	38 (33)
Musculoskeletal disorders (arthrosis, back pain, osteoporosis, scoliosis)	20 (17)
Endocrine disorders (hypothyroidism, Hashimoto thyroiditis, diabetes)	14 (12)
Respiratory diseases (asthma, COPD, pulmonary hypertension)	11 (10)
Gastrointestinal diseases (Crohn’s disease, colitis ulcerosa, diverticular disease, gastritis)	8 (7)
Neurological disorders (epilepsy, multiple sclerosis, stroke, seizures)	7 (6)
Hematologic/coagulation disorders (Factor V Leiden, von Willebrand disease, thrombocytopenia, MDS/MPN)	5 (4)
Dermatological diseases (psoriasis, vitiligo, rosacea)	4 (3)
Renal diseases (chronic kidney disease, renal insufficiency)	4 (3)
Autoimmune/systemic diseases (SLE)	2 (2)
Oncological diseases (breast cancer, prostate cancer, BCC)	4 (3)
Other conditions	6 (5)

This table summarizes comorbidities reported by 82 of the 116 study participants (71%) in addition to hereditary hemorrhagic telangiectasia. Comorbidities were collected using open-ended responses and subsequently grouped by disease category. Multiple responses were possible; therefore, individual patients may be represented in more than one category. Data are presented as number of patients and percentage of the total study population

Abbreviations: GI = gastrointestinal; CV = cardiovascular; COPD = chronic obstructive pulmonary disease; MDS/MPN = myelodysplastic syndromes and myeloproliferative neoplasms; BCC = basal cell carcinoma; SLE = systemic lupus erythematosus

At least one additional comorbidity besides HHT was reported by 71% of the patients (*N* = 82/116), 29% reported no comorbid conditions (*N* = 34/116), while psychiatric comorbidities were reported by 18% (*N* = 21/116). The most frequently reported disease groups were cardiovascular conditions (*N* = 38/116, 33%), musculoskeletal disorders (*N* = 20/116, 17%), endocrine disorders (*N* = 14/116, 12%), and respiratory diseases (*N* = 11/116, 10%; Table 2).

Most patients suffered from epistaxis (*N* = 110/116, 97%) with a mean ESS of 5.3 (SD: ±2.5), and 38% of respondents were diagnosed with gastrointestinal involvement (*N* = 43/116). Pulmonary arteriovenous malformations were identified in 48 patients (*N* = 48/116, 42%); hepatic in 26 (*N* = 26/116, 23%), and six patients had cerebral vascular malformations (*N* = 6/116, 6%). Half of the participants were anemic with a mean hemoglobin level of 11.8 g/dl (± SD 2.6 g/dl, *N* = 58/111, 50%). Facial telangiectasias were present in 86% (*N* = 100/116), as determined by self-report or clinical documentation. Of these, 39% (*N* = 45/116) had undergone at least one laser treatment for facial telangiectasias. The TSI was available for 85% of the participants (*N* = 98/116). The disease severity was classified as mild in 64% (*N* = 63/98), moderate in 28% (*N* = 27/98), and severe in 8% (*N* = 8/98). An officially recognized degree of disability (GdB) was reported by 44% patients (*N* = 51/116). The GdB is assigned considering all underlying conditions. When categorized by severity, 27% of the patients (*N* = 31/116) had a moderate degree of disability (GdB 30–50), while 9% (*N* = 10/116) had a severe degree of disability (GdB ≥ 60). Patients with comorbidities had a higher risk of severe GdB compared to those without comorbidities (OR = 4.47, 95% CI 1.75–11.41, *p* = 0.002). Disease severity as measured by the TSI, was not reflected by the reported GdB (OR = 1.00, 95% CI 0.89–1.13, *p* = 0.992). PROMIS-29+2 domain T-scores ranged from 45.2 ± 8.9 to 56.7 ± 8.0 across all assessed domains. Mean scores for physical function (45.2 ± 8.9) and ability to participate in social roles (45.6 ± 8.9) were below the population reference mean of 50, whereas higher scores were observed for anxiety (56.7 ± 8.0), depression (56.3 ± 8.2), fatigue (56.0 ± 9.8), and sleep disturbance (53.7 ± 8.6) in HHT. Mean pain intensity on a scale from 0 to 10 was 2.8 ± 2.4 (Table 1).

In response to the question “*Do you think you look pale or tired due to your low hemoglobin level?”,* 67% (*N* = 72/108) of respondents answered *“yes,”* indicating a perception of visible signs of anemia. Thirty-two patients (34.4%) said that they don’t suffer currently from an anemia. Of those patients, 6 (18.8%) feel that they look pale or tired because of their low hemoglobin level. Conversely, one third (*N* = 36/116, 33%) did not observe such signs. When asked “*Do you consider them disturbing or unattractive?”,* 68% (*N* = 68/116) patients reported finding their telangiectasias cosmetically bothersome, while 32% (*N* = 32/116) were not disturbed by these lesions. Participant characteristics are summarized in Table 1.

### FACE-Q scores

FACE-Q subscale scores for the total cohort and stratified by sex are shown in Table 3. In the total study population, the lowest scores were recorded for “appearance-related psychological distress” and “satisfaction with facial appearance overall”, both below 50. Women and men showed comparable FACE-Q scores across all subscales.

**Table 3:** FACE-Q subscale scores for all participants

Subscale (mean SD)	Total group (N = 116)	Women (N = 72)	Men (N = 44)	p-value
FACE-Q Social function	57.2 (20.7)	56.3 (20.8)	58.3 (20.1)	0.835
FACE-Q Psychological function	59.0 (20.2)	58.5 (20.5)	59.1 (19.3)	0.741
FACE-Q Satisfaction with Facial Appearance Overall	49.4 (17.3)	47.8 (16.6)	51.5 (18.2)	0.430
FACE-Q Appearance-related psychological distress	30.5 (21.2)	30.5 (20.4)	30.7 (22.5)	0.862
FACE-Q Satisfaction with nostrils	68.5 (28.7)	67.5 (29.2)	59.5 (28.2)	0.718
FACE-Q Satisfaction with nose	65.9 (23.0)	65.5 (23.1)	66.1 (23.0)	0.760

Data show the results of the Facial Assessment and Cosmetic Enhancement Quality of life Questionnaire (Face-Q) subscale scores for the total study population and stratified by sex. Values are presented as mean ± standard deviation. FACE-Q scores range from 0 to 100, with higher scores indicating better outcomes, except for the appearance-related psychological distress scale, where higher scores indicate greater distress. *p*-values refer to comparisons between women and men

### Linear regression analyses

Univariate analyses of demographic, clinical, and patient-reported variables in relation to FACE-Q outcomes are shown in supplementary Table S2, indicating that mainly psychological burden, symptom severity, and PROMIS domains were associated with FACE-Q scores.

Multivariate linear regression analyses are presented in Table 4 (including only variables with *p* < 0.05) and in Supplementary Table S3 (full multivariate regression model). Psychological and functional PROMIS domains emerged as the primary independent predictors of QoL and satisfaction with appearance. In contrast, objective clinical parameters, such as hemoglobin level, anemia status, and facial telangiectasias did not demonstrate significant associations with any outcome after adjustment for covariates.

**Table 4:** Multivariate linear regression analyses of the FACE-Q subscales and patient-reported variables in HHT

Questionnaire	Variable	ß (95% CI Upper – Lower)	p-Value
FACE-Q Subscale Social Function	Do you consider your facial telangiectasias disturbing or unattractive?	−9.51 (−18.04; −0.99)	**0.029**
	PROMIS Ability to Participate in Social Roles and Activities	1.34 (0.59; 2.10)	**<0.001**
FACE-Q Subscale Satisfaction with Facial Appearance	Smoking yes/no	−11.80 (−1.60; −22.01)	**0.024**
	PROMIS Physical Function	0.53 (1.03; 0.02)	**0.041**
	PROMIS Fatigue	−0.58 (−0.12; −1.04)	**0.014**
FACE-Q Subscale Appearance-Related Psychological Distress	Do you think you look pale or tired due to your low hemoglobin levels?	−9.09 (−0.94; −17.23)	**0.029**
	Do you consider your facial telangiectasias disturbing or unattractive?	−9.33 (−2.16; −16.51)	**0.012**
	PROMIS Sleep Disturbance	−0.52 (−0.09; −0.95)	**0.019**
FACE-Q Subscale Satisfaction with Nose	Do you consider your facial telangiectasias disturbing or unattractive?	−17.39 (−28.94; −5.84)	**0.004**

HHT = hereditary hemorrhagic telangiectasia; PROMIS = Patient-Reported Outcomes Measurement Information System; FACE-Q = a rigorously developed patient-reported outcome measure. Only predictors with *p* < 0.05 are presented in this table; the complete multivariate regression models are provided in Supplementary Table S3. No significant predictors (*p* < 0.05) were identified for the Psychological Function and Satisfaction with Nostrils subscales in the multivariate regression analyses. Therefore, these subscales are not presented in this table

For the FACE-Q Social Function domain, increased participation in social roles and activities were significantly associated with higher social confidence (ß_adjusted_ = 1.3,4 95% CI [0.59; 2.09], *p* < 0.001). In contrast, perceiving facial telangiectasias as unattractive was associated with lower social function ß_adjusted_ = −9.51, 95% CI [−18.04; −0.99], *p* = 0.029). No other variables, including psychiatric comorbidity, fatigue, or epistaxis severity, exhibited significant independent effects.

In the FACE-Q Psychological Function model, elevated anxiety levels were significantly associated with reduced psychological well-being (ß_adjusted_ = −0.90, 95% CI [−1.53; −0.26], *p* = 0.006).

Smoking was negatively associated with facial appearance satisfaction (ß_adjusted_ = −11.80, 95% CI [−22.01; −1.60], *p* = 0.024). Better physical functioning correlated positively with satisfaction (ß_adjusted_ = 0.53, 95% CI [0.02; 1.03], *p* = 0.041), while increased fatigue was associated with lower satisfaction (ß_adjusted_ = −0.58, 95% CI [−1.04; −0.12], *p* = 0.014).

Appearance-related psychological distress was significantly greater among individuals perceiving themselves as pale or tired due to low hemoglobin levels (adjusted β = −9.09, 95% CI −17.23 to −0.94, *p* = 0.029). Sleep disturbance also emerged as an independent negative factor associated with greater appearance-related psychological distress (adjusted β = −0.52, 95% CI −0.95 to −0.09, *p* = 0.019). In addition, perceiving facial telangiectasias as aesthetically disturbing was independently associated with multiple FACE-Q outcomes, including greater appearance-related psychological distress (adjusted β = −9.33, 95% CI −16.51 to −2.16, *p* = 0.012), lower social function (adjusted β = −9.51, 95% CI −18.04 to −0.99, *p* = 0.029), and lower satisfaction with the nose (adjusted β = −17.39, 95% CI −28.94 to −5.84, *p* = 0.004). The results of the multivariable linear regression analyses are presented in Table 4.

## Discussion

In this study, we examined appearance-related QoL in people affected by HHT using validated PROMs, focusing on facial telangiectasias and subjective appearance perception. Our findings demonstrate that appearance-related QoL in HHT is primarily driven by psychosocial factors and individual perceptions, rather than by objective disease parameters or overall disease severity. Multivariate analyses identified psychological symptom burden, physical functioning, social participation, and subjective appearance perception as strong and independent predictors of FACE-Q outcomes. Consistently, participants who perceived themselves as pale or tired due to low hemoglobin levels, and those who found their facial telangiectasias disturbing, reported greater impairment in daily life. Self-reported perception of facial telangiectasias as disturbing remained independently associated, supporting a perception-driven rather than treatment-driven explanation. By contrast, hematologic parameters (including hemoglobin levels and anemia status (yes/no)) and overall disease severity, as assessed by the ESS and TSI were not independently associated with appearance-related QoL.

HHT symptoms typically increase with age [24]. The mean age of our cohort was relatively high (60 ± 14 years), and a substantial proportion of participants were retired. Although retirement is associated with reduced work-related social interaction [25, 53], age was not independently associated with social functioning as assessed by the FACE-Q. These findings indicate that impairments in social participation are more likely attributable to disease-related psychosocial factors rather than age or employment status alone.

The WHO definitions of anemia were used to ensure comparability with previous HHT studies, while acknowledging ongoing discussions regarding sex-specific hemoglobin thresholds.(54) Sex-stratified analyses were conducted, given prior evidence from studies of iron-deficiency anemia demonstrating greater negative impacts on self-image and QoL in women, particularly in relation to visible symptoms such as pallor and hair loss [26, 27]. In addition, female hormones have been proposed to influence disease expression in HHT [28], and women have been reported to exhibit higher rates of hepatic vascular malformations [2]. However, no sex-specific differences were observed in FACE-Q outcomes or other clinical characteristics in the present study. Hemoglobin levels and anemia prevalence were comparable between men and women. Notably, although women were younger on average, most were postmenopausal. As menopause typically occurs between 45 and 55 years of age (mean ~51 years), after which menstruation-related blood loss ceases [26, 27], this age distribution likely contributed to the lack of sex-related differences in appearance-related QoL.

In addition to HHT, 71% of participants reported comorbidities, including neurological and dermatological disorders, which may contribute to perceived stigma or impaired appearance-related QoL [29–37]. However, comorbidities were not independently associated with FACE-Q outcomes. Similarly, GdB, which intended to reflect the cumulative impact of all underlying conditions [17], was higher in patients with comorbidities but did not correlate with HHT severity according to the TSI. This indicates that current disability assessments primarily capture multimorbidity rather than HHT-specific disease burden, underscoring the need for improved objective measures of disease impact in HHT.

Smoking and sleep disturbances are known to negatively affect the perception of facial skin and appearance [38, 39]. In Germany in 2024, approximately 22% of the general population were smokers [40]. In this cohort, 8% of participants were current smokers. This low prevalence may indicate greater health awareness and risk-avoidant behavior among individuals with chronic multimorbidity [41]. In contrast, impaired sleep quality was common and associated with a more negative perception of facial appearance [51]. Sleep disturbances in HHT may arise from multiple factors, including restless legs syndrome [42], nocturnal epistaxis, and the psychological burden managing a chronic hereditary disease [9, 43]. Nevertheless, these mechanisms remain speculative, because sleep quality in the present study was not assessed using objective diagnostic methods.

### Study limitations

Several limitations should be acknowledged. Psychological burden was assessed exclusively using PROMs without formal psychiatric evaluation, and facial telangiectasias were evaluated based on subjective perception rather than objective quantification. The FACE-Q was developed to assess patient-reported outcomes in individuals undergoing facial aesthetic procedures and has subsequently been adapted and validated for several facial conditions, including craniofacial disorders, facial skin cancer, and facial paralysis. However, it has not been validated specifically in patients with hereditary hemorrhagic telangiectasia. [12, 18, 19] Although FACE-Q and PROMIS are well-validated instruments, they do not fully capture all functional or contextual aspects of disease burden [44–46]. In addition, the cross-sectional design precludes causal inference. However, these findings underscore the importance of incorporating patients’ subjective perception into clinical care, particularly in psychologically burdensome conditions such as HHT. Addressing psychosocial factors may help maintain quality of life, prevent worsening of depressive and anxiety symptoms, and ultimately reduce both individual and healthcare-related burden.

## Conclusion

The findings of our study emphasize the importance of a patient-centered approach that actively addresses appearance-related concerns, perceived stigma, and social participation among individuals with HHT. Incorporating parts of validated PROMs, such as the FACE-Q (especially questions regarding satisfaction of facial appearance and resulting psychological distress) and PROMIS instruments (particularly questions focusing on physical function, the ability to participate in social roles, anxiety depression, fatigue, and sleep disturbance), into clinical routine assessments may facilitate the identification of individuals at risk for psychosocial distress who might otherwise be overlooked based on clinical parameters alone. Based on our findings, future studies should analyze modified patient-centred symptom-based questionnaire for individuals with HHT. Longitudinal designs are necessary to evaluate how changes in disease manifestations, treatment strategies, and psychosocial support influence appearance-related quality of life over time. In addition, qualitative studies may provide deeper insight into how patients interpret and manage visible disease features, thereby informing targeted counseling strategies and multidisciplinary care models to enhance overall well-being in HHT [47–54].

## Electronic supplementary material

Below is the link to the electronic supplementary material.


Supplementary Material 1



Supplementary Material 2



Supplementary Material 3



Supplementary Material 4


## Data Availability

The datasets generated and/or analyzed during the current study are not publicly available due to privacy and ethical restrictions but are available from the corresponding author on reasonable request.

## Abbreviations

BMI: Body Mass Index
CI: Confidence Interval
ESS: Epistaxis Severity Score
FACE-Q: Facial Aesthetic Patient-Reported Outcome instrument
GdB: Degree of Disability (Grad der Behinderung)
HHT: Hereditary Hemorrhagic Telangiectasia
FACE-Q: Facial Assessment and Cosmetic Enhancement Quality of Life Questionnaire
PROMIS-29+2: Patient-Reported Outcomes Measurement Information System 29-Item Profile with Two Cognitive Function Items
QoL: Quality of Life
SD: Standard Deviation
TSI: Toronto Severity Index
