# Supplementary material for: Impact of facial telangiectasias and anemia on quality of life in patients with hereditary hemorrhagic telangiectasia: a cross-sectional study using FACE-Q
Source: J Patient Rep Outcomes. 2026 Aug 26;10:160. doi: 10.1186/s41687-026-01179-x (PMC13530060; doi:10.1186/s41687-026-01179-x)
Supplement: Supplementary file 1 [file 41687_2026_1179_MOESM1_ESM.pdf]

Supplementary Figure S1 General questionnaire

Please answer all questions and mark the option that best applies. If you have additional information you would like to share, please write it on the back of the page.

1. Sex: ____________________
2. Age: ____________________
3. Height: ____________________
4. Weight: ____________________
5. Do you smoke? If yes, since when and how much per day?
6. How often do you drink alcohol?
   Never / 1–2× per week / >2× per week / Daily
   Which type of alcohol do you drink most frequently?
   Beer / Wine / Spirits (e.g., schnapps, vodka)
7. a) What is your occupation? ____________________
   b) Do you have customer/patient contact? No / Yes
8. Do you have a certified disability?
   No / Yes, degree: ________ Is this due to HHT (Osler disease)? No / Yes
9. Relationship status:
   Single / In a relationship / Prefer not to say
10. Have you ever received psychological or psychiatric treatment?
   No / Yes / Prefer not to say
11. Do you currently have a psychological disorder?
   No / Yes, which (e.g., depression): ____________________
12. Do you have any other medical conditions?
   No / Yes, which: ____________________
13. Have you noticed telangiectasia (small dilated blood vessels appearing as red dots) on your face?
   No / Yes
14. If yes, do you perceive them as disturbing or unattractive?
   No / Yes
15. Have the telangiectasia ever been treated?
   No / Yes, how often and how (e.g., laser): ____________________
16. Did the treatment of telangiectasia result in visible scarring?
   No / Yes
17. How often do you experience nosebleeds?
   <1× per month / 1× per month / 1× per week / >1× per week / 1× per day / >1× per day
18. How long does your nosebleed usually last?
   <1 minute / 1–5 minutes / 6–15 minutes / 16–30 minutes / >30 minutes
19. How would you usually describe the intensity of your nosebleeds?
   Rather mild / dripping | Rather heavy / flowing
20. Have you sought medical assistance because of your nosebleeds?
   No / Yes
21. Do you currently suffer from anemia (low hemoglobin level)?
   No / Yes / I don't know
22. a) Have you ever received a blood transfusion?
   No / Yes, due to nosebleeds / Yes, due to gastrointestinal bleeding / Yes, due to both
   Yes, for another reason: ____________________
   b) Approximately how many blood transfusions in total? __________
23. Do you feel that you look pale or tired because of your low hemoglobin level?
   No / Yes
24. Are you taking medication due to anemia / iron deficiency anemia (e.g., iron tablets)?
   No / Yes, which and dosage: ____________________
