# Supplementary material for: Impact of facial telangiectasias and anemia on quality of life in patients with hereditary hemorrhagic telangiectasia: a cross-sectional study using FACE-Q
Source: J Patient Rep Outcomes. 2026 Aug 26;10:160. doi: 10.1186/s41687-026-01179-x (PMC13530060; doi:10.1186/s41687-026-01179-x)
Supplement: Supplementary file 2 [file 41687_2026_1179_MOESM2_ESM.docx]

**Supplemental Material**

**Table S1: Information on the occupation of patients with HHT**

| **Occupation category** | **N (%)** |
| --- | --- |
| Retired (incl. early retirement) | 52 (45) |
| Employed – administrative / office / commercial | 19 (16) |
| Healthcare professionals | 14 (12) |
| Care professions (elderly & childcare) | 6 (5) |
| Management / leadership positions | 7 (6) |
| Technical / engineering professions | 6 (5) |
| IT professions | 4 (3) |
| Homemaker / household-related work | 6 (5) |
| Students | 2 (2) |
| Education / social work / psychology / clergy | 5 (4) |
| Service / hospitality / marketing / creative | 6 (5) |
| Public service / civil servants | 4 (3) |
| Insurance / finance professions | 3 (3) |
| Occupational status was based on self-reported profession and grouped according to the primary functional role. Employment categories reflect the main occupational activity at the time of assessment.  For analyses involving occupational exposure to interpersonal interaction, professions were additionally classified according to the presence or absence of regular customer or client contact. This classification was based on the typical duties associated with the reported profession rather than job titles alone. As such, some occupational categories (e.g. management or administrative roles) may include individuals both with and without regular customer contact.  In the total cohort (number of patients (N) = 116), 41 patients (35%) reported having regular customer contact, 20 patients (17%) were classified as having no regular customer contact, 52 patients (45%) were retired (including early retirement or disability), and 3 patients (3%) had missing data. No participants reported being unemployed or actively seeking employment. | |
