# Supplementary material for: Impact of facial telangiectasias and anemia on quality of life in patients with hereditary hemorrhagic telangiectasia: a cross-sectional study using FACE-Q
Source: J Patient Rep Outcomes. 2026 Aug 26;10:160. doi: 10.1186/s41687-026-01179-x (PMC13530060; doi:10.1186/s41687-026-01179-x)
Supplement: Supplementary file 3 [file 41687_2026_1179_MOESM3_ESM.docx]

**Supplementary Table S2. Univariate linear regression analyses of demographic, clinical, and patient-reported variables and FACE-Q subscale scores**

| **Questionnaire** | **Variable** | **ß (95% CI Upper- Lower)** | **P-Value** |
| --- | --- | --- | --- |
| **FACE-Q Subscale**  **Social Function** | Age [years] | 0.03 (-0.23 ; 0.31) | 0.773 |
|  | Gender (0=men, 1= women) | -0.07 (-10.94 ; 4.67) | 0.428 |
|  | Body Mass Index [kg/m2] | 0.13 (-0.18 ; 1.08) | 0.161 |
|  | Smoking yes/no | -0.04 (-16.60 ; 10.53) | 0.659 |
|  | Profession | -0.03 (-4.95 ; 3.44) | 0.722 |
|  | Relationship status | 0.16 (-2.00 ; 19.89) | 0.108 |
|  | Comorbidity yes/no | -8.77 (-16.92 ; -0.62) | **0.035** |
|  | Psychiatric comorbidity yes/no | 0.35 (-27.08 ; -8.91) | **< 0.001** |
|  | Hemoglobulin level [g/dl] | 0.09 (-0.81 ; 2.14) | 0.371 |
|  | Anemia based on Hb level  (women <12g/dl, men <13g/dl) | -0.01 (-8.24 ; 7.08) | 0.880 |
|  | Presence of facial telangiectasias | -0.05 (-14.69 ; 8.26) | 0.580 |
|  | Treatment for facial telangiectasias | -0.87 (-7.28 ; 9.02) | 0.833 |
|  | Epistaxis Severity Scale | -0.30 (-11.16 ; -2.76) | **< 0.001** |
|  | Toronto Severity Index | -0.57 (-1.80 ; 0.66) | 0.383 |
|  | Do you think you look pale or tired due to your low hemoglobin levels? Yes/no | -13.14 (-20.93 ; -5.34) | **< 0.001** |
|  | Do you consider your facial telangiectasias disturbing or unattractive? Yes/no | -12.42 (-20.84 ; -4.02) | 0.004 |
|  | PROMIS Physical Function | 0.41 (0.56 ; 1.35) | **< 0.001** |
|  | PROMIS Anxiety | -0.46 (-1.62 ; -0.78) | **< 0.001** |
|  | PROMIS Depression | -0.49 (-1.65 ; -0.84) | **< 0.001** |
|  | PROMIS Fatigue | -0.44 (-1.30 ; -0.60) | **< 0.001** |
|  | PROMIS Sleep Disturbance | -0.34 (-1.22 ; -0.38) | **< 0.001** |
|  | PROMIS Ability to Participate in Social Roles and Activities | 0.54 (0.90 ; 1.64) | **< 0.001** |
|  | PROMIS Pain Interference | -0.27 (-0.99 ; -0.19) | **0.004** |
|  | PROMIS Cognitive Function | 0.25 (0.22 ; 1.24) | **0.005** |
|  | PROMIS Pain intensity | -0.19 (-3.10 ; -0.02) | **0.047** |
| **FACE-Q Subscale**  **Psychological Function** | Age [years] | 0.46 (-0.19 ; 0.33) | 0.618 |
|  | Gender (0=men, 1=women) | 0.04 (-9.26 ; 5.97) | 0.670 |
|  | Body Mass Index [kg/m2] | 0.09 (-0.33 ; 0.92) | 0.348 |
|  | Smoking yes/no | -0.17 (-24.57 ; 1.28) | **0.077** |
|  | Profession | -0.07 (-5.66 ; 2.49) | 0.442 |
|  | Relationship status | 0.11 (-4.58 ; 17.48) | 0.249 |
|  | Comorbidity yes/no | -4.06 (-12.13 ; 4.02) | 0.322 |
|  | Psychiatric comorbidity yes/no | -0.36 (-27.02 ; -9.36) | **< 0.001** |
|  | Hemoglobulin level [g/dl] | 0.18 (-0.05 ; 2.80) | **0.059** |
|  | Anemia based on Hb level  (women <12g/dl, men <13g/dl) | -0.12 (-12.07 ; 2.85) | 0.224 |
|  | Presence of facial telangiectasias | -0.055 (-14.51 ; 7.92) | 0.562 |
|  | Treatment for facial telangiectasias | -1.39 (-9.40 ; 6.61) | 0.732 |
|  | Epistaxis Severity Index | -0.44 (-13.51 ; -5.84) | **< 0.001** |
|  | Toronto Severity Index | 0.40 (-0.81 ; 1.60) | **0.517** |
|  | Do you think you look pale or tired due to your low hemoglobin levels? | -18.0 (-25.23 ; -10.77) | **< 0.001** |
|  | Do you consider your facial telangiectasiasdisturbing or unattractive? | -12.61 (-20.84 ; -4.38) | **0.003** |
|  | PROMIS Physical Function | 0.46 (0.67 ; 1.42) | **< 0.001** |
|  | PROMIS Anxiety | -0.64 (-1.97 ; -1.27) | **< 0.001** |
|  | PROMIS Depression | -0.62 (-1.87 ; -1.16) | **< 0.001** |
|  | PROMIS Fatigue | -0.57 (-1.49 ; -0.87) | **< 0.001** |
|  | PROMIS Sleep Disturbance | -0.46 (-1.45 ; -0.68) | **< 0.001** |
|  | PROMIS Ability to Participate in Social Roles and Activities | 0.58 (0.97 ; 1.66) | **< 0.001** |
|  | PROMIS Pain Interference | -0.40 (-1.23 ; -0.49) | **< 0.001** |
|  | PROMIS Cognitive Function | 0.19 (0.02 ; 1.03) | **0.041** |
|  | PROMIS Pain intensity | -0.28 (-3.74 ; -0.81) | **0.003** |
| **FACE-Q Subscale**  **Satisfaction with Facial Appearance** | Age [years] | 0.03 (-0.19 ; 0.26) | 0.789 |
|  | Gender (0=men, 1= women) | -0.12 (-10.87 ; 2.11) | 0.184 |
|  | Body Mass Index [kg/m2] | 0.16 (-0.07 ; 0.99) | **0.088** |
|  | Smoking yes/no | -0.317 (-30.03 ; -8.48) | **< 0.001** |
|  | Profession | -0.03 (-3.99 ; 2.85) | 0.740 |
|  | Relationship status | 0.03 (-7.66 ; 10.63) | 0.748 |
|  | Comorbidity yes/no | 0.27 (-6.73 ; 7.28) | 0.939 |
|  | Psychiatric comorbidity yes/no | -0.10 (-12.26 ; 3.70) | 0.291 |
|  | Hemoglobulin level [g/dl] | 0.18 (-0.05 ; 2.44) | **0.060** |
|  | Anemia based on Hb level  (women <12g/dl, men <13g/dl) | -0.10 (-10.08 ; 2.95) | 0.281 |
|  | Presence of facial telangiectasias, self-reported | -0.10 (-14.68 ; 4.23) | 0.276 |
|  | Treatment for facial telagiectasias | -3.93. (-10.54 ; 2.68) | 0.241 |
|  | Epistaxis Severity Index | -0.37 (-10.17 ; -3.50) | **< 0.001** |
|  | Do you think you look pale or tired due to your low hemoglobin levels? | -17.72 (-23.63 ; -11.81) | **< 0.001** |
|  | Do you consider your facial telangiectasiasdisturbing or unattractive? | -13.74 (-20.38 ; -7.11) | **< 0.001** |
|  | PROMIS Physical Function | 0.43 (0.52 ; 1.17) | **< 0.001** |
|  | PROMIS Anxiety | -0.43 (-1.30 ; -0.06) | **< 0.001** |
|  | PROMIS Depression | -0.43 (-1.26 ; -0.56) | **< 0.001** |
|  | PROMIS Fatigue | -0.54 (-1.24 ; -0.69 | **< 0.001** |
|  | PROMIS Sleep Disturbance | -0.42 (-1.16 ; -0.49) | **< 0.001** |
|  | PROMIS Ability to Participate in Social Roles and Activities | 0.46 (0.58 ; 1.24) | **< 0.001** |
|  | PROMIS Pain Interference | -0.36 (-1.00 ; -0.35) | **< 0.001** |
|  | PROMIS Cognitive Function | 0.17 (-0.04 ; 0.83) | **0.074** |
|  | PROMIS Pain intensity | -0.32 (-3.43 ; -1.00) | **< 0.001** |
| **FACE-Q Subscale**  **Appearance-Related Psychological Distress** | Age [years] | -0.05 (-0.35 ; 0.20) | 0.578 |
|  | Gender (0=men, 1= women) | 0.02 (-7.20 ; 8.81) | 0.843 |
|  | Body Mass Index [kg/m2] | 0.01 (-0.64 ; 0.68) | 0.949 |
|  | Smoking yes/no | 0.13 (-3.87 ; 23.61) | 0.157 |
|  | Profession | 0.05 (-3.19 ; 5.62) | 0.586 |
|  | Relationship status | -0.21 (-23.67 ; -1.30) | **0.029** |
|  | Comorbidity yes/no | 6.48 (-2.01 ; 14.97) | 0.133 |
|  | Psychiatric comorbidity yes/no | 0.28 (5.56 ; 24.95) | **0.002** |
|  | Hemoglobulin level [g/dl] | -0.10 (-2.36 ; 0.70) | 0.283 |
|  | Anemia based on Hb level  (women <12g/dl, men <13g/dl) | 0.01 (-7.67 ; 8.24) | 0.943 |
|  | Presence of facial telangiectasias | 0.08 (-6.83 ; 17.08) | 0.398 |
|  | Treatment for facial telangiectasias | 1.99 (-6.45 ; 10.45) | 0.640 |
|  | Epistaxis Severity Score | 0.41 (5.77 ; 14.36) | **< 0.001** |
|  | Toronto Severity Index | 0.50 (-0.77 ; 1.78) | 0.434 |
|  | Do you think you look pale or tired due to your low hemoglobin levels? | 15.07 (6.98 ; 23.16) | **< 0.001** |
|  | Do you consider your facial telangiectasias disturbing or unattractive? | 11.95 (3.32 ; 20.73) | **0.008** |
|  | PROMIS Physical Function | -0.42 (-1.40 ; -0.60) | **< 0.001** |
|  | PROMIS Anxiety | 0.59 (1.16 ; 1.94) | **< 0.001** |
|  | PROMIS Depression | 0.54 (0.98 ; 1.78) | **< 0.001** |
|  | PROMIS Fatigue | 0.48 (0.70 ; 1.40) | **< 0.001** |
|  | PROMIS Sleep Disturbance | 0.49 (0.80 ; 1.60) | **< 0.001** |
|  | PROMIS Ability to Participate in Social Roles and Activities | -0.47 (-1.51 ; -0.72) | **< 0.001** |
|  | PROMIS Pain Interference | 0.37 (0.45 ; 1.24) | **< 0.001** |
|  | PROMIS Cognitive Function | -0.32 (-1.45 ; -0.43) | **< 0.001** |
|  | PROMIS Pain intensity | 0.29 (0.93 ; 4.03) | **0.002** |
| **FACE-Q Subscale**  **Satisfaction with Nostrils** | Age [years] | -0.13 (-0.63 ; 0.11) | 0.173 |
|  | Gender (0=men, 1= women) | -0.05 (-13.49 ; 8.10) | 0.622 |
|  | Body Mass Index [kg/m2] | -0.021 (-0.99 ; 0.79) | 0.826 |
|  | Smoking yes/no | -0.11 (-29.56 ; 7.71) | 0.248 |
|  | Profession | 0.02 (-5.33 ; 6.38) | 0.860 |
|  | Relationship status | -0.14 (-27.59 ; 3.93) | 0.140 |
|  | Comorbidity yes/no | -2.50 (-14.16 ; 9.16) | 0.672 |
|  | Psychiatric comorbidity yes/no | 0.06 (-9.58 ; 17.96) | 0.548 |
|  | Hemoglobulin level [g/dl] | 0.11 (-0.81 ; 3.25) | 0.236 |
|  | Anemia based on Hb level  (women <12g/dl, men <13g/dl) | -0.09 (-15.35 ; 5.74) | 0.369 |
|  | Presence of facial telangiectasias | -0.003 (-16.60 ; 16.14) | 0.978 |
|  | Treatment for facial telangiectasias | 0.87 (-10.66 ; 12.40) | 0.881 |
|  | Epistaxis Severity Index | -0.21 (-12.84 ; -0.66) | **0.030** |
|  | Toronto Severity Index | -0.92 (-2.63 ; 0.80) | 0.292 |
|  | Do you think you look pale or tired due to your low hemoglobin levels? | -8.83 (-20.48 ; 2.80) | 0.135 |
|  | Do you consider your facial telangiectasias disturbing or unattractive? | -17.74 (-29.63 ; -5.86) | **0.004** |
|  | PROMIS Physical Function | 0.31 (0.42 ; 1.55) | **< 0.001** |
|  | PROMIS Anxiety | -0.12 (-1.10 ; 0.21) | 0.180 |
|  | PROMIS Depression | -0.19 (-1.26 ; -0.01) | **0.046** |
|  | PROMIS Fatigue | -0.33 (-1.47 ; -0.46) | **< 0.001** |
|  | PROMIS Sleep Disturbance | -0.23 (-1.33 ; -0.15) | **0.014** |
|  | PROMIS Ability to Participate in Social Roles and Activities | 0.30 (0.38 ; 1.53) | **0.001** |
|  | PROMIS Pain Interference | -0.26 (-1.36 ; -0.26) | **0.004** |
|  | PROMIS Cognitive Function | 0.10 (-0.34 ; 1.10) | 0.294 |
|  | PROMIS Pain intensity | -0.18 (-4.24 ; 0.05) | **0.055** |
| **FACE-Q Subscale**  **Satisfaction with Nose** | Age [years] | -0.003 (-0.30 ; 0.29) | 0.972 |
|  | Gender (0=men, 1= women) | -0.03 (-10.17 ; 7.13) | 0.729 |
|  | Body Mass Index [kg/m2] | 0.04 (-0.56 ; 0.87) | 0.666 |
|  | Smoking yes/no | -0.15 (-26.60 ; 3.23) | 0.124 |
|  | Profession | -0.13 (-7.66 ; 1.51) | 0.186 |
|  | Relationship status | 0.01 (-12.00 ; 13.27) | 0.921 |
|  | Comorbidity yes/no | 0.18 (-0.53 ; 18.25) | **0.051** |
|  | Psychiatric comorbidity yes/no | 0.05 (-8.31 ; 13.56) | 0.635 |
|  | Hemoglobulin level [g/dl] | 0.10 (-0.72 – 2.41) | 0.286 |
|  | Anemia based on Hb level  (women <12g/dl, men <13g/dl) | -0.07 (-11.29 ; 4.99) | 0.445 |
|  | Presence of facial telangiectasias | 0.13 (-4.19 ; 21.60) | 0.184 |
|  | Treatment for facial telangiectasias | -9.71 (-18.45 ; -0.97) | **0.030** |
|  | Epistaxis Severity Index | -0.18 (-9.61 ; 0.32) | **0.066** |
|  | Toronto Severity Index | 0.30 (-1.08 ; 1.67) | 0.671 |
|  | Do you think you look pale or tired due to your low hemoglobin levels? | -9.72 (-18.88 ; -0.56) | **0.038** |
|  | Do you consider your facial telangiectasias disturbing or unattractive? | -20.20 (-28.96 ; -11.44) | **< 0.001** |
|  | PROMIS Physical Function | 0.20 (0.04 ; 0.97) | **0.032** |
|  | PROMIS Anxiety | -0.20 (-1.09 ; -0.06) | **0.030** |
|  | PROMIS Depression | -0.22 (-1.11 ; -0.11) | **0.018** |
|  | PROMIS Fatigue | -0.30 (-1.13 ; -0.30) | **< 0.001** |
|  | PROMIS Sleep Disturbance | -0.29 (-1.25 ; -0.29) | **0.002** |
|  | PROMIS Ability to Participate in Social Roles and Activities | 0.23 (0.12 ; 1.07) | **0.014** |
|  | PROMIS Pain Interference | -0.17 (-0.88 ; 0.02) | **0.061** |
|  | PROMIS Cognitive Function | 0.13 (-0.17 ; 0.99) | 0.160 |
|  | PROMIS Pain intensity | -0.19 (-3.47 ; -0.05) | **0.044** |
| Univariate linear regression analyses examining associations between demographic, clinical, and patient-reported variables and results of the Facial Assessment and Cosmetic Enhancement Quality of life Questionnaire (FACE-Q). Beta (β) coefficients with 95% confidence intervals (CI) and p-values are shown. P-values below 0.10 in bold as they will be analyzed further in multivariate regression analysis. Negative β values indicate lower FACE-Q scores with increasing predictor values. FACE-Q scores range from 0 to 100, with higher scores indicating better outcomes, except for the appearance-related psychological distress scale, where higher scores indicate greater distress. PROMIS T-scores are standardized to a mean of 50 and a standard deviation of 10.  TSI = Toronto Severity Index (categories: mild, moderate, severe); ESS = Epistaxis Severity Scale (0-1 no epistaxis, mild severity 1-4, moderate severity 4-7, severe epistaxis 7-10); PROMIS = Patient-Reported Outcomes Measurement Information System, in this case the PROMIS 29+2 questionnaire was implemented.  Hb = hemoglobulin level  Gender : (0=men, 1= women)  Profession (with costumer contact, without costumer contact, pensioner)  Relationship status: single versus in a relationship | | | |
