# Supplementary material for: Impact of facial telangiectasias and anemia on quality of life in patients with hereditary hemorrhagic telangiectasia: a cross-sectional study using FACE-Q
Source: J Patient Rep Outcomes. 2026 Aug 26;10:160. doi: 10.1186/s41687-026-01179-x (PMC13530060; doi:10.1186/s41687-026-01179-x)
Supplement: Supplementary file 4 [file 41687_2026_1179_MOESM4_ESM.docx]

**Supplementary Table S3. Multivariate linear regression analyses of the FACE-Q subscales and patient-reported variables in HHT**

| **Questionnaire** | **Variable** | **ß (95% CI Upper – Lower )** | **P-Value** |
| --- | --- | --- | --- |
| **FACE-Q Subscale**  **Social Function** | Intercept | -2.94 (-92.12; 86.23) | 0.948 |
|  | Comorbidity yes/no | -2.44 (-12.15; 7.27) | 0.618 |
|  | Psychiatric comorbidity yes/no | -5.22 (-15.59; 5.15) | 0.319 |
|  | Do you think you look pale or tired due to your low hemoglobin levels? | -3.26 (-13.55; 7.04) | 0.530 |
|  | **Do you consider your facial telangiectasias disturbing or unattractive?** | **-9.51 (-18.04; -0.99)** | **0.029** |
|  | Epistaxis severity score | 3.50 (-3.26; 10.26) | 0.305 |
|  | PROMIS Physical Function | 0.12 (-0.56; 0.81) | 0.723 |
|  | PROMIS Anxiety | -0.11 (-0.85; 0.63) | 0.771 |
|  | PROMIS Depression | -0.41 (-1.24; 0.43) | 0.337 |
|  | PROMIS Fatigue | 0.44 (-0.20; 1.08) | 0.170 |
|  | PROMIS Sleep Disturbance | -0.23 (-0.77; 0.30) | 0.385 |
|  | **PROMIS Ability to Participate in Social Roles and Activities** | **1.34 (0.59; 2.10)** | **<0.001** |
|  | PROMIS Pain Interference | 0.12 (-0.76; 0.99) | 0.793 |
|  | PROMIS Cognitive Function | 0.08 (-0.48; 0.64) | 0.768 |
|  | PROMIS Pain intensity | 1.49 (-1.52; 4.49) | 0.326 |
| **FACE-Q Subscale**  **Psychological Function** | Intercept | 151.00 (68.88 ; 233.13) | <0.001 |
|  | Smoking yes/no | -5.83 (5.31 ; -16.96) | 0.300 |
|  | Psychiatric comorbidity yes/no | -4.68 (4.38 ; -13.74) | 0.306 |
|  | Hemoglobulin level [g/dl] | 4.48 (12.44 ; -3.48) | 0.265 |
|  | Epistaxis severity score | -4.83 (1.62 ; -11.28) | 0.139 |
|  | Do you think you look pale or tired due to your low hemoglobin levels? | -4.15 (4.78 ; -13.09) | 0.357 |
|  | Do you consider your facial telangiectasias disturbing or unattractive? | -2.81 (5.01 ; -10.63) | 0.476 |
|  | PROMIS Physical Function | -0.04 (0.57 ; -0.64) | 0.904 |
|  | **PROMIS Anxiety** | **-0.90 (-0.26 ; -1.53)** | **0.006** |
|  | PROMIS Depression | -0.08 (0.64 ; -0.80) | 0.927 |
|  | **PROMIS Fatigue** | **-0.34 (0.24 ; -0.92)** | 0.249 |
|  | PROMIS Sleep Disturbance | -0.21 (0.27 ; -0.68) | 0.389 |
|  | PROMIS Ability to Participate in Social Roles and Activities | 0.22 (0.89 ; -0.46) | 0.519 |
|  | PROMIS Pain Interference | 0.35 (1.11 ; -0.41) | 0.361 |
|  | PROMIS Cognitive Function | -0.40 (0.07 ; -0.88) | 0.096 |
|  | PROMIS Pain intensity | -0.47 (2.07 ; -3.01) | 0.710 |
| **FACE-Q Subscale**  **Satisfaction with Facial Appearance** | Intercept | 65.11 (-9.31 ; 139.52) | 0.086 |
|  | Body Mass Index [kg/m2] | 0.17 (0.81 ; -0.46) | 0.592 |
|  | **Smoking yes/no** | **-11.80 (-1.60 ; -22.01)** | **0.024** |
|  | Hemoglobulin level [g/dl] | -0.08 (1.17 ; -1.32) | 0.905 |
|  | Epistaxis severity score | -0.09 (4.67 ; -4.85) | 0.970 |
|  | **PROMIS Physical Function** | **0.53 (1.03 ; 0.02)** | **0.041** |
|  | PROMIS Anxiety | -0.31 (0.22 ; -0.85) | 0.245 |
|  | PROMIS Depression | 0.28 (0.87 ; -0.31) | 0.353 |
|  | **PROMIS Fatigue** | **-0.58 (-0.12 ; -1.04)** | **0.014** |
|  | PROMIS Sleep Disturbance | **-0.36 (0.06 ; -0.78)** | 0.090 |
|  | PROMIS Ability to Participate in Social Roles and Activities | 0.06 (0.63 ; -0.50) | 0.828 |
|  | PROMIS Pain Interference | 0.39 (1.05 ; -0.28) | 0.253 |
|  | PROMIS Cognitive Function | -0.18 (0.24 ; -0.59) | 0.398 |
|  | PROMIS Pain intensity | -0.95 (1.21 ; -3.11) | 0.383 |
| **FACE-Q Subscale**  **Appearance-Related Psychological Distress** | Intercept | 89.02 (15.46 ; 162.59) | 0.019 |
|  | Relationship versus Single | -1.34 (8.10 ; -10.78) | 0.778 |
|  | Psychiatric comorbidity | 5.27 (13.55 ; -3.01) | 0.208 |
|  | Epistaxis severity score | 3.62 (9.11 ; -1.86) | 0.191 |
|  | **Do you think you look pale or tired due to your low hemoglobin levels?** | **-9.09 (-0.94 ; -17.23)** | **0.029** |
|  | **Do you consider your facial telangiectasias disturbing or unattractive?** | **-9.33 (-2.16 ; -16.51)** | **0.012** |
|  | PROMIS Physical Function | 0.18 (0.73 ; -0.38) | 0.527 |
|  | PROMIS Anxiety | -0.17 (0.44 ; -0.79) | 0.576 |
|  | PROMIS Depression | 0.11 (0.81 ; -0.60) | 0.761 |
|  | PROMIS Fatigue | -0.31 (0.22 ; -0.84) | 0.241 |
|  | **PROMIS Sleep Disturbance** | **-0.52 (-0.09 ; -0.95)** | **0.019** |
|  | PROMIS Ability to Participate in Social Roles and Activities | 0.15 (0.77 ; -0.46) | 0.621 |
|  | PROMIS Pain Interference | 0.05 (0.75 ; -0.66) | 0.897 |
|  | PROMIS Cognitive Function | -0.03 (0.45 ; -0.51) | 0.899 |
|  | PROMIS Pain intensity | -0.54 (1.96 ; -3.03) | 0.668 |
| **FACE-Q Subscale**  **Satisfaction with Nostrils** | Intercept | 54.56 (-87.54 ; 196. 66) | 0.447 |
|  | Epistaxis severity score | 3.94 (14.08 ; -6.20) | 0.441 |
|  | Do you consider your facial telangiectasias disturbing or unattractive? | -10.27 (3.68 ; -24.22) | 0.147 |
|  | PROMIS Physical Function | 0.14 (1.19 ; -0.90) | 0.785 |
|  | PROMIS Anxiety | 0.95 (2.14 ; -0.25) | 0.118 |
|  | PROMIS Depression | 0.09 (1.37 ; -1.19) | 0.888 |
|  | PROMIS Fatigue | -0.30 (0.65 ; -1.25) | 0.532 |
|  | PROMIS Sleep Disturbance | -0.49 (0.34 ; -1.33) | 0.243 |
|  | PROMIS Ability to Participate in Social Roles and Activities | 0.99 (2.22 ; -0.24) | 0.112 |
|  | PROMIS Pain Interference | -1.05 (0.34 ; -2.44) | 0.136 |
|  | PROMIS Cognitive Function | -0.12 (0.76 ; -1.00) | 0.785 |
|  | PROMIS Pain intensity | 3.28 (8.14 ; -1.58) | 0.183 |
| **FACE-Q Subscale**  **Satisfaction with Nose** | Intercept | 49.09 (-65.34; 163.52) | 0.395 |
|  |  |  |  |
|  | Epistaxis severity score | -0.46 (-9.44; 8.51) | 0.918 |
|  | Treatment for facial telangiectasias | -2.74 (-13.83; 8.34) | 0.623 |
|  | Do you think you look pale or tired due to your low hemoglobin levels? | 0.35 (-13.12; 13.82) | 0.959 |
|  | **Do you consider your facial telangiectasias disturbing or unattractive?** | **-17.39 (-28.94; -5.84)** | **0.004** |
|  | PROMIS Physical Function | -0.01 (-0.91; 0.90) | 0.992 |
|  | PROMIS Anxiety | 0.46 (-0.50; 1.42) | 0.345 |
|  | PROMIS Depression | -0.32 (-1.39; 0.75) | 0.551 |
|  | PROMIS Fatigue | -0.15 (-0.98; 0.68) | 0.723 |
|  | PROMIS Sleep Disturbance | -0.46 (-1.14; 0.22) | 0.178 |
|  | PROMIS Ability to Participate in Social Roles and Activities | 0.47 (-0.50; 1.44) | 0.341 |
|  | PROMIS Pain Interference | 0.66 (-0.47; 1.78) | 0.247 |
|  | PROMIS Cognitive Function | 0.00 (-0.71; 0.71) | 0.995 |
|  | PROMIS Pain intensity | -3.05 (-6.93; 0.83) | 0.121 |
| HHT = hereditary hemorrhagic telangiectasia, PROMIS = Patient-Reported Outcomes Measurement Information System, FACE-Q =is a rigorously developed patient-reported outcome measure | | | |
